# Psychobiological Factors Affecting Cortisol Variability in Human-Dog Dyads

**DOI:** 10.1371/journal.pone.0170707

**Published:** 2017-02-08

**Authors:** Iris Schöberl, Manuela Wedl, Andrea Beetz, Kurt Kotrschal

**Affiliations:** 1 Department of Behavioural Biology, University of Vienna, Vienna, Austria; 2 Core Facility Konrad Lorenz Research Station, University of Vienna, Grünau im Almtal, Austria; 3 Department of Special Education, Institut für sonderpädagogische Entwicklungsförderung und Rehabilitation, University of Rostock, Rostock, Germany; University of Marburg, GERMANY

## Abstract

Stress responses within dyads are modulated by interactions such as mutual emotional support and conflict. We investigated dyadic psychobiological factors influencing intra-individual cortisol variability in response to different challenging situations by testing 132 owners and their dogs in a laboratory setting. Salivary cortisol was measured and questionnaires were used to assess owner and dog personality as well as owners' social attitudes towards the dog and towards other humans. We calculated the individual coefficient of variance of cortisol (iCV = sd/mean*100) over the different test situations as a parameter representing individual variability of cortisol concentration. We hypothesized that high cortisol variability indicates efficient and adaptive coping and a balanced individual and dyadic social performance. Female owners of male dogs had lower iCV than all other owner gender-dog sex combinations (*F* = 14.194, *p<*0.001), whereas owner Agreeableness (NEO-FFI) scaled positively with owner iCV (*F* = 4.981, *p* = 0.028). Dogs of owners high in Neuroticism (NEO-FFI) and of owners who were insecure-ambivalently attached to their dogs (FERT), had low iCV (*F* = 4.290, *p* = 0.041 and *F* = 5.948, *p* = 0.016), as had dogs of owners with human-directed separation anxiety (RSQ) or dogs of owners with a strong desire of independence (RSQ) (*F* = 7.661, *p* = 0.007 and *F* = 9.192, *p* = 0.003). We suggest that both owner and dog social characteristics influence dyadic cortisol variability, with the human partner being more influential than the dog. Our results support systemic approaches (i.e. considering the social context) in science and in counselling.

## Introduction

One of the core issues in social relationships is stress coping [1]. For example, socio-positive interactions between dyadic partners may dampen physiological stress responses and promote relaxation [2,3] as a result of mutual emotional support [4–6]. Consequently, social animals show better recovery from aversive experiences when in the company of supportive partners than when alone [7]. On the other hand, social conflicts are among the most powerful stressors [8–10].

It has been shown that emotional social support also exists between humans and dogs [4,11–13]. This may be explained by shared and evolutionary conservative social mechanisms, including brain and stress physiology [14–18]. Dogs have socially adapted to live with humans for more than 30 000 years [19–24] and therefore, are closely adapted to human social performance. For example, dogs recognize and integrate emotional information from humans [25–29] and adjust their behavior to human communication signals [13] and human emotions [30,31]. There may even be emotional contagion between owners and dogs [32,33].

Stress coping, i.e. the behavioral, physiological and mental effort to master challenging situations [34] differs between individuals [35,36]. This may also be because mental representations influence the individual validation of potential stressors [18]. In fact, inter-individual variability in coping styles seems to be adaptive [37]. We suggest that parallel to heart rate variability [38], intra-individual cortisol variability reflects the adaptive flexibility of the hypothalamic-pituitary-adrenal (HPA) axis to respond to a variable environment [39]. This is based on findings that decreased cortisol variability is related to a down-regulated HPA axis by chronic stress [39], to bad health status, moderate well-being [40,41] and limited executive functions [42]. Hence, intra-individual cortisol variability over standardized situations may be an informative measure of stress coping, with a high amplitude between arousal peaks and relaxation lows reflecting healthy regulation.

Individual differences in glucocorticoid variability are modulated genetically and epigenetically as well as by social experience [36,37,43]. Comparatively little is known about psychobiological factors influencing intra-individual variability in glucocorticoid response to different challenging social situations. Men emphasize different coping strategies than women, focusing on “fight and flight” instead of “tend and befriend”, which is the strategy preferred by females (Kudielka & Kirschbaum 2005). Men and women also differ in their interactions with, and attitudes towards, other animals [44]. This may also affect interaction styles with animals and the behavioral responses of these animals. For example, dogs seem generally more relaxed when petted by females than males [45]. Furthermore, male dogs of male owners are more sociable and active than male dogs of female owners [46] and male dogs of male owners had the lowest cortisol concentration compared to all other owner gender-dog sex combinations during an experimental assessment of their attachment [47]. This may be related to different behavior in humans depending on the gender of the interaction partner [48–51]. For example, same-gender pairs perform better during a computer task and show more verbal interactions than mixed-gender pairs [49]. Trust seems to be greater within same-gender pairs, than mixed-gender pairs [50]. However, other studies show that competition is greater and cooperation less pronounced within same-gender pairs than mixed-gender pairs [48,51]. With the shared social mechanisms of humans and dogs in mind, we therefore suggest that owner gender-dog sex combination may affect dyadic human-dog interactions and thereby affect stress coping.

Gender is also linked to personality. For example, women score higher in Neuroticism (NEO-FFI dimension 1) [52–54] and Agreeableness (NEO-FFI dimension 4) [53], whereas men score higher in Extraversion (NEO-FFI dimension 2) [54]. Personality is connected to stress coping as well [34]. Humans high in Conscientiousness (NEO-FFI dimension 5), Agreeableness and Extraversion cope better with challenges in their lives than humans low on these dimensions [55]. Furthermore, Neuroticism is related to low expectations towards being supported [55] and to increased anxiety [55,56]. Interestingly, owner Neuroticism was found to be linked to low cortisol concentration in dogs [47,57].

A considerable proportion of individual HPA variability may be linked to relationships with partners, with distinct roots in early life history. Securely attached children [58,59] respond behaviorally and physiologically adequately to challenging situations and will quickly down-regulate behavioral and physiological responses after successful coping [60]. Along the lines of the parent-offspring model of human-dog relationships [57,61], the same principles also seem to work between dogs and their owners: We found that dogs classified as securely attached to their owner had a smaller cortisol increase during an attachment assessment and play situations with their owner than insecurely attached dogs [47]. Based on the same data set, we presently asked whether psychobiological factors influence intra-individual cortisol variability in owners as well as in their dogs.

We hypothesized that high intra-individual cortisol variability is an indicator of efficient and adaptive coping, because a flexible HPA axis supports adequate responses to a variable environment, e.g. high cortisol variability is related to good health status and well-being [39–41]. Therefore, we predict that dyadic parameters which facilitate socio-positive interactions are related to high cortisol variability, e.g. owners with secure relationship patterns towards their dogs, but also towards other humans, may have dogs with high cortisol variability. Agreeable or extraverted owners are expected to show high cortisol variability, because of their competent stress coping. As male dogs of female owners are less sociable and relaxed than male dogs of male owners [46] we predict low cortisol variability over different challenging situations for male dogs and their female owners compared to all other owner gender-dog sex combinations.

## Materials and Methods

To investigate the influence of psychobiological factors on owner and dog intra-individual cortisol modulation, we tested human-dog dyads in a laboratory setting and measured their cortisol concentration before and after different challenging social situations.

The current study follows a pilot study with only male dogs with their male or female owners [46,57,62] and formed part of an interdisciplinary project that investigated human-dog relationship and attachment. In previous papers [47,63] part of the methods we used were already described in detail and so we only report the overall design of the study and methods not sufficiently described elsewhere, and ask readers to refer to our previous papers for more details where applicable.

### Ethical approval

Participation in our study was voluntary; dog owners were informed that they could stop the test situation at any time and also signed a written information and consent form. Data collection was conducted according to the standards of the Code of Ethics of the World Medical Association (Declaration of Helsinki), the EU Directive 2010/63/EU for animal experiments and Uniform Requirements for manuscripts submitted to Biomedical journals and the German Society of Psychology (Ethische Richtlinien der DGPs und des BDP). Ethical review for this study was conducted by the animal-welfare committee of the Faculty of Life Sciences, University of Vienna (approval number: 2014–015). Ethical approvement for the human participation in our study was granted by the Ethics Commission of the DGPs (Deutsche Gesellschaft für Psychologie, approval number AB 07_2011).

### Subjects and general procedure

We collected data from 132 human-dog dyads. Intact pet dogs (mean age ± SD: 3.95 ± 1.83 years; mean weight ± SD: 29.53 ± 13.19 kg) and their primary attachment figure, the “owner” (mean age ± SD: 43.76 ± 10.71 years) were tested. All dogs had been adopted by their owners as puppies (mean age ± SD: 9.77 ± 3.50 weeks). Owner gender-dog sex combination was counterbalanced (35 female owners with female dogs, 35 male owners with male dogs, 31 female owners with male dogs and 31 male owners with female dogs). Different dog breeds and mixed breeds participated, but were limited to no more than three dogs of the same pure breed per owner gender-dog sex combination.

Owner-dog dyads participated in two test sessions. A third session was scheduled with a subsample of 59 dyads [47], but is not considered in the present paper. The two sessions took about 90 minutes each and were scheduled in a test room at the University of Vienna. At the beginning of both sessions the general procedure was explained, which took about ten minutes. A camcorder (Canon Legria-HF-G10) with a wide-angle conversion lens (Canon WD-H58W) was fixed on the wall of the test room to videotape the two sessions. An experimenter observed the procedure from outside the room via a monitor, which was connected to the camcorder. During the first session, the focus was on the behavior of dog and owner in response to a novel room, separation from each other and reunion afterwards as well as to a challenging task. The aim of the second session was the measurement of heart rate and observation of behavior during a play situation and during two threatening situations. All tests are explained below. Furthermore, the owners completed questionnaires and took saliva samples from themselves and their dog during the sessions (Table 1). In addition, owners collected saliva samples during one non-test control day from themselves and their dog.

**Table 1:** Time schedule of the first and second session.

Session	Kind of Task	Description of Task	Duration [min]
**1**	E	Initial phase	10
	S	Saliva Samples 1	2
	T	Dog Alone in Novel Room	3
	T	Picture Viewing Test	8
	Q	Questionnaire	15
	S	Saliva Samples 2	2
	T	Challenge Task	10
	Q	Questionnaire	15
	S	Saliva Samples 3	2
**2**	E	Initial phase	10
	S	Saliva Samples 1	2
	T	Attaching Polar-harness	5
	T	Playing for Adaptation	5
	T	Resting for Adaptation	5
	Q	Questionnaire	15
	S	Saliva Samples 2	2
	T	Threat 1	3
	Q	Questionnaire	15
	S	Saliva Samples 3	2
	T	Threat 2	3
	Q	Questionnaire	15
	S	Saliva Samples 4	2

E, explaining the general procedure; S, saliva sampling; T, tests; Q, questionnaires.

### Questionnaires

Owners completed questionnaires to assess human and dog personality, owner-dog relationship, as well as owner-dog interaction style and demographics. The questionnaires and the scales, which were revealed via principal component analysis (PCA), are the same as used in a previous study [47].

- The NEO Five-Factor Inventory is a 60-item questionnaire designed to measure personality in adult humans in five domains: Neuroticism, Extraversion, Openness, Agreeableness, and Conscientiousness [52,64].
- The Monash Canine Personality Questionnaire (MCPQ-R) [65] was translated to German in cooperation with a bilingual expert. A principal component analysis (PCA) from the translated questionnaire revealed five reliable axes (reliability via Cronbach-Alpha—given in the same order as the axes: 0.809, 0.837, 0.794, 0.777, 0.712): “Active-excitable”, “Obedient-reliable”, “Insistent-goal directed”, “Nervous-anxious” and “Cool-friendly”.
- The Relationship Scales Questionnaire (RSQ) [66,67] captures different aspects of attachment via the scales “Separation anxiety”, “Closeness anxiety”, “Lack of trust” and “Wish to be independent”, which are associated with adult interpersonal relationship patterns [68].
- The FERT (Fragebogen zu Erfahrungen mit Tieren) is a questionnaire in German on the relationship with companion animals [69]. The questionnaire was adapted for dog owners. PCA revealed four reliable axes (reliability via Cronbach-Alpha—given in the same order as the axes: 0.817, 0.812, 0.745, 0.624): “Dog as social supporter” and “Communication”, “Secure-caregiving” and “Insecure-ambivalent”.
- Our self-developed interaction questionnaire included questions on owner interaction style towards the dog during daily life and revealed two reliable axes via PCA (reliability via Cronbach-Alpha—given in the same order as the axes: 0.773, 0.681): “Affirmative interaction style” (e.g. positive reinforcement such as giving treats, play, and verbal praise) and “Aversive interaction style” (e.g. positive punishment such as leash-jerk, telling the dog off, shaking the dog’s neck).

### Saliva samples

Saliva samples were taken from owners and dogs to measure cortisol concentration. It is known that blood cortisol concentrations peak approximately 20 minutes after a dog encounters a stressor [45] and 20–40 minutes after a human encounters a stressor, depending on the duration and stressfulness of the task [70]. Thus, saliva samples from both owners and their dogs were taken before the tests and 15 minutes after the end of each test. Both sessions were scheduled at similar times in the afternoon within each dyad, mainly to avoid potential morning peaks in cortisol concentration [71]. Additionally, owners were asked to take saliva samples in the morning (8 am), at noon (2pm) and in the evening (8pm) during one resting day without testing (the calmest day of the week), between the first and second session to obtain baseline values.

The experimenter instructed the owner how to take saliva samples from themselves and their dog at the beginning of the first session; all samples were taken by the owner himself/herself. Salimetrics oral swabs were used to obtain saliva samples from the owners and Salimetrics children's swabs for the dogs`samples. First the owner put the oral swab under his/her tongue and then he/she took the sample from the dog at the same time; the children's swab was inserted in the dog’s cheek pouch for about 90 seconds. Before taking the saliva sample the dog’s salivation was stimulated by smelling cheese. Although it is known that cheese does not interfere with measuring cortisol [72] the dog received a piece of cheese only after the saliva sample was taken. Each sample was frozen at -20°C (Sarstedt tubes) until analysis. Cortisol concentration was analyzed by enzyme immunoassay (EIA) [73].

### Tests

#### Picture viewing test

This test aims to collect information about the dog-human relationship and orientation toward each other. To activate the attachment system, the owner was asked to leave the dog alone in the novel room for three minutes immediately after entering the room. During this time the owner and experimenter (IS) observed the dog via the monitor outside the room. Then the owner entered the room again and looked at 14 dog pictures placed on the walls and windows. The dog was allowed to move freely within the room; the owner was asked to behave towards the dog as he/she normally would. During this test the experimenter was outside the room observing owner and dog via the monitor. The experimenter came back into the room as soon as the owner finished the task, or after eight minutes.

#### Challenge task

The Challenge task aims to collect information on how owner and dog cope with practical tasks. The owner was asked to lead the dog as efficiently and safely as possible over a wire mesh bridge (height 0.6 m, length 5 m). Then the owner was asked to lead the dog onto a wobbly podium where the dog should remain for at least five seconds. The dyads were given four minutes to complete each of these tasks. Finally, the owner was asked to give the dog the commands “sit” and “lie down” with a maximum of two repetitions for each command. The owners were asked to interact with their dog as they usually do during daily life; owners were free to give the commands verbally, via gesture or both and were also allowed to use food if this was also part of the daily routine.

#### Adaptation to heart rate monitor

The owner was asked to attach heart rate monitors to him/herself and his/her dog. This took about five minutes. Then the owner was asked to play with the dog for five minutes to adapt to the heart rate monitor. Toys as well as treats could be used. Afterwards the dog was on leash for five minutes and was asked by the owner to sit or lie down, but not to move. Heart rate and heart rate variability were measured from owners and dogs, but will be published separately and the technical details are described in another paper [63].

#### Mild threat

Two counterbalanced randomly ordered threat situations, one test with and one without the owner present, were conducted. For safety reasons, the dog was tethered to the wall with a leash during both threats. For the “mild threat with the owner present” the owner was instructed to behave in the same way he/she would do in similar situations in daily life. While the “mild threat without the owner present” was conducted, the owner could observe the situation from outside via a monitor. During both tasks the experimenter (IS) observed from outside to interrupt if necessary. The threat was conducted by another female experimenter, the “threatening person”, who wore a long black coat with a hood and a ski mask; just the eyes of the threatening person were visible.

After entering the room, the threatening person knocked at the door from inside to get the dog’s attention. Then the threatening person stared at the dog and moved towards it. After each step the threatening person stopped for three seconds and kept staring at the dog. After the third step the threatening person turned away and left the room at the end of the first threat. The same procedure was followed for the second threat, but this time the situation was resolved at the end: the threatening person turned away from the dog, moved to the corner opposite to the dog, removed the ski mask and coat and approached the dog in a friendly manner while talking and offering it pieces of cheese. Then the formerly threatening person left the room and the dog was let off the leash. This test was described in detail in a former paper [47].

### Data analysis

Cortisol data were checked for outliers across the entire sample size of 132 dyads. Differences were found in absolute cortisol concentration as well as in standard deviations of cortisol concentration in male owners taking medication (n = 8) and male owners without medication (n = 58). Therefore, we excluded cortisol concentration values of those eight owners under medication from further analysis. Neither hormonal contraception nor medication use by women influenced cortisol concentrations. Cortisol concentration values of one dog were excluded from further analysis because of pseudo-pregnancy. Afterwards the remaining cortisol data were corrected for outliers by taking the mean cortisol value of all dog samples and separately, of all human samples, except the morning samples during the resting day. Error correction was done separately for the morning samples during the resting day, because humans exhibit a circadian pattern of cortisol with a cortisol peak in the morning [74, 75] and also in dogs, circadian patterns are found with differing morning cortisol concentrations [76–79]. All values higher than three standard deviations of the mean were excluded from further analysis [80], which was the case in 1.6% of all cortisol concentration values for dogs and in 2.3% of all cortisol concentration values for owners.

The mean salivary cortisol concentration value ± standard deviation for session one and two over all owners was 6.85 ± 5.01 ng/ml; over all dogs 2.33 ± 2.71 ng/ml. The mean cortisol maximum ± standard deviation for session one and two over all owners was 10.75 ± 6.18 ng/ml; over all dogs 4.66 ± 4.05 ng/ml. The coefficient of variation of cortisol for each individual (iCV = sd/mean*100) was taken as an indicator of intra-individual cortisol variability in dogs and owners. Therefore, for each owner and each dog the mean value and standard deviation was calculated over all cortisol samples from the first and second session as a base for iCV. Only individuals with at least four applicable cortisol concentration values after error correction were taken into account for the calculation of the iCV. This resulted in 116 dogs (87.9%) and 115 owners (87.1%) being included in the analysis of iCV. A maximum of seven values was possible for owners and for dogs to calculate the iCV (mean number of values for iCV ± SD: Owners = 6.86 ± 0.51, Dogs = 6.55 ± 0.77).

Statistical analysis was performed with SPSS 21 software. The Shapiro-Wilk Test was used to test for normal distribution. Data were not normally distributed; thus, nonparametric tests (Friedman, Mann-Whitney U, Wilcoxon and Spearman’s rank correlation) were used for univariate statistics. General Linear Models (GLM) were calculated for owner iCV and dog iCV. As owner and dog iCV was not normally distributed a log transformation was conducted to gain normally distributed data. Owner personality (NEO-FFI), dog personality (MCPQ-R), owner to dog attachment (FERT), owner relationship to other humans (RSQ), owner interaction style towards the dog (interaction questionnaire), as well as owner and dog age, were tested as covariates. Owner gender-dog sex combination was included as a factor. We selected these explanatory variables as main effects and removed them in order of decreasing significance (if p>0.1). Terms with p<0.1 were retained in the final model; to ensure that excluded terms did not explain the variance they were re-entered one by one into the final model [81]. Only terms with *p*<0.05 were considered a significant influence on the dependent variable. All significances (*p*<0.05) are given two-tailed.

As described in a previous paper [47], alpha correction for multiple comparisons was not considered here because this generally increases the risk of type-II error at a comparatively low potential of decreasing type-I error [82]. Instead, Cohen’s effect size was calculated for univariate statistics, with d = 0.2 indicating a small effect, d = 0.5 a medium effect and d = 0.8 a large effect [83]. Because non-significant results do not mean that there is no effect [84] we also include tendencies (*p*<0.1) within this paper.

## Results

Univariate analysis was done separately for owner gender, because owner gender differences were found for absolute owner and dog cortisol concentration: Female owners and their dogs had higher morning cortisol concentration during the day without testing than male owners and their dogs (MWU: owners: n = 110, *Z* = -1.958, *p* = 0.05, d = 0.38; dogs: n = 111, *Z* = -2.843, *p* = 0.004, d = 0.56). Male owners had higher cortisol concentration during the second session than female owners (MWU: sample1: n = 115, *Z* = -3.322, *p* = 0.001, d = 0.652; sample2: n = 113, *Z* = -3.474, *p* = 0.001, d = 0.692; sample3: n = 115, *Z* = -3.467, *p* = 0.001, d = 0.683; sample4: n = 114, *Z* = -3.482, *p*<0.001, d = 0.69). Dogs of female owners had higher cortisol concentration after the second threat (last sample of the second session; MWU: n = 102, *Z* = -1.995, *p* = 0.046, d = 0.403) and by trend after the picture viewing test (second sample during the first session; MWU: n = 120, *Z* = -1.75, *p* = 0.08, d = 0.324) than dogs of male owners. No differences were found related to dog sex.

When threat data were analyzed according to owner presence, male owners had higher cortisol concentration values after the threat with and threat without the owner present than female owners (MWU: threat with: n = 114, *Z* = -3.260, *p* = 0.001, d = 0.641; threat without: n = 113, *Z* = -3.693, *p*<0.001, d = 0.741). Dogs of female owners had higher cortisol concentration values after the threat without owner present than dogs of male owners (MWU: n = 107, *Z* = -2.046, *p* = 0.041, d = 0.404). No differences were found depending on whether the owner was present or not during the threat situation and whether it was the first or second threat.

### Changes in cortisol concentration

**Fig 1:**
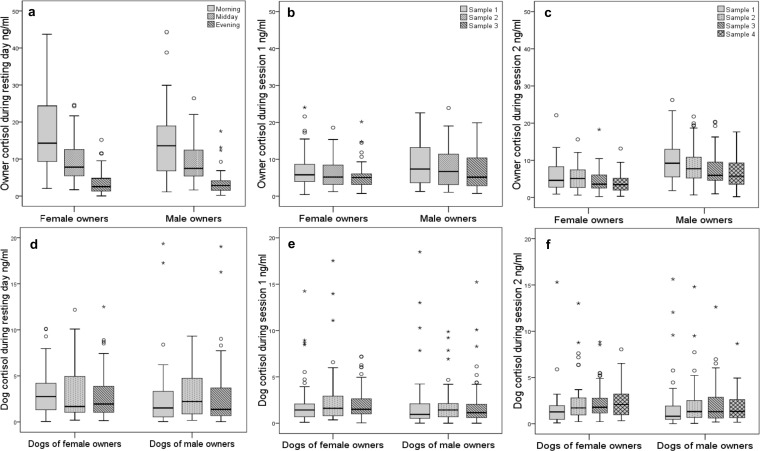
Changes in cortisol in owners and dogs. Cortisol concentration during the resting day, during the first session and second session in owners (a, b, c) and their dogs (d, e, f). Median, interquartile range, upper and lower whiskers and outliers are given.

As expected due to the usual diurnal pattern of cortisol, owner cortisol decreased from the morning sample until the evening sample during the resting day (Friedman: female owners: n = 58, *χ*^2^ = 81.759, *df* = 2, *p*<0.001; male owners: n = 49, *χ*^2^ = 48.810, *df* = 2, *p*<0.001). Also from the first to the last sample during both sessions cortisol decreased in owners (Friedman: female owners: session 1: n = 62, *χ*^2^ = 22.871, *df* = 2, *p*<0.001; session 2: n = 60, *χ*^2^ = 41.554, *df* = 3, *p*<0.001; male owners: session 1: n = 54, *χ*^2^ = 16.926, *df* = 2, *p*<0.001; session 2: *n* = 49, *χ*^2^ = 38.748, *df* = 3, *p*<0.001). In dogs of female owners, cortisol increased from the first to the last sample during the second session (Friedman: n = 41, *χ*^2^ = 10.978, *df* = 3, *p* = 0.012), whereas this was neither found for dogs of male owners nor during the first session for dogs of male and female owners (Fig 1).

In owners and dogs, cortisol concentration changed during the challenges in a relatively complex way (Table 2). Also, results at the group level did not show a clear cortisol pattern for different challenging tasks. Instead, differences were found at the individual level; some decreased and some increased in cortisol. Those who showed an increase in one situation did not necessarily show an increase in another situation. Therefore, to further analyze which factors influenced intra-individual cortisol variability the coefficient of variance for each individual (iCV) was calculated over the different challenging situations (Picture viewing test, bridge/podium, play, threat with and without the owner present); with the rationale that a high iCV would reflect an adaptive/optimal HPA regulation, whereas a low iCV would indicate a somewhat constrained condition.

**Table 2:** Cortisol increase and decrease in owners and dogs during the different challenging situations.

Task	Subject	Owner gender	% decrease	% increase	Wilcoxon *Z*	Wilcoxon *p*	Cohen's d
Picture Viewing	Owner	Female	64.5	35.5	-1.953	0.051	**0.512**
		Male	61.1	38.9	-1.537	0.124	**0.428**
	Dog	Female	33.9	66.1	-2.064	**0.039**	**0.574**
		Male	48.3	51.7	-0.155	0.877	0.040
Challenge Task	Owner	Female	69.8	30.2	-3.156	**0.002**	**0.867**
		Male	74.5	25.5	-3.226	**0.001**	**0.966**
	Dog	Female	49.1	50.9	-1.201	0.230	**0.328**
		Male	45.8	54.2	-0.03	0.976	0.007
Play	Owner	Female	57.4	42.6	-1.281	0.200	**0.333**
		Male	66.7	33.3	-2.034	**0.042**	**0.594**
	Dog	Female	32.0	68.0	-3.403	**0.001**	**1.098**
		Male	34.0	66.0	-1.598	0.110	**0.446**
Threat 1	Owner	Female	70.0	30.0	-3.327	**0.001**	**0.942**
		Male	69.2	30.8	-3.490	**<0.001**	**1.120**
	Dog	Female	50.0	50.0	-0.222	0.824	0.064
		Male	49.1	50.9	-0.075	0.940	0.020
Threat 2	Owner	Female	71.4	28.6	-3.516	**<0.001**	**0.998**
		Male	63.5	36.5	-2.559	**0.010**	**0.768**
	Dog	Female	44.2	55.8	-0.604	0.546	0.183
		Male	57.7	42.3	-0.674	0.500	0.188

Cortisol concentration values before and after the tests were compared. Significances p<0.05 and effect sizes d>0.2 are given in bold.

### Intra-individual cortisol variability (iCV)

For testing whether a high or low iCV was related to different cortisol patterns, individuals were divided into two groups: iCV higher or lower than median iCV. Dogs of male owners had higher iCV values than dogs of female owners (MWU: n = 116, *Z* = -2.123, *p* = 0.034, d = 0.402); thus, dog iCV was separately analyzed for owner gender. No owner gender difference for iCV was found in humans; iCV did not differ according to sex, neither in humans nor in dogs.

Owners with a high iCV had lower absolute cortisol concentration during both sessions than owners with a low iCV (MWU: first session: sample 2: n = 112, *Z* = -2.011, *p* = 0.044, d = 0.387; sample 3: n = 114, *Z* = -1.981, *p* = 0.048, d = 0.378; second session: sample 1: n = 113, *Z* = -2.544, *p* = 0.011, d = 0.493; sample 2: n = 112, *Z* = -2.610, *p* = 0.009, d = 0.509; sample 3: n = 112, *Z* = -3.616, *p*<0.001, d = 0.727; sample 4: n = 111, *Z* = -4.260, *p*<0.001, d = 0.884). Owners with a high iCV were more likely to have a decrease in cortisol during the picture viewing test (MWU: n = 110, *Z* = -2.204, *p* = 0.027, d = 0.43). Dogs with a high iCV and a female owner had lower cortisol concentration at the beginning of the second session than dogs with a low iCV and a female owner (MWU: n = 53, *Z* = -1.983, *p* = 0.047, d = 0.566). Dogs with a high iCV and a male owner were more likely to have a cortisol decrease during the second threat and thus had lower cortisol concentration after the second threat than dogs with a low iCV and a male owner (MWU: n = 51, *Z* = -2.480, *p* = 0.013, d = 0.741 and *Z* = -2.158, *p* = 0.032, d = 0.634).

#### Factors influencing intra-individual cortisol variability in owners and their dogs

**Fig 2:**
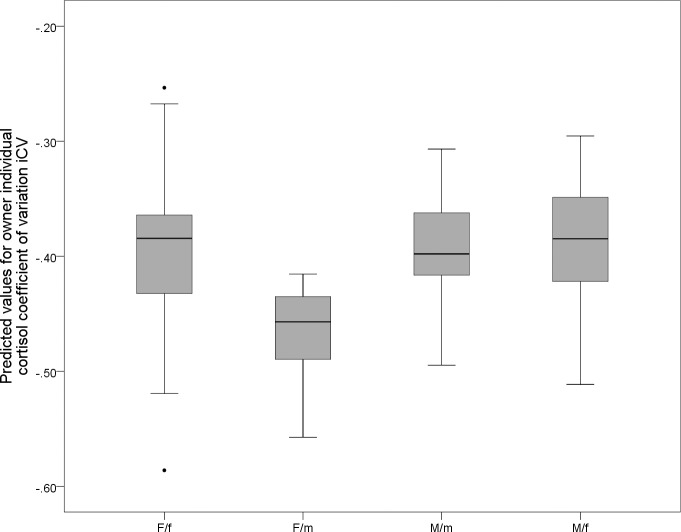
Owner individual cortisol coefficient of variation (iCV) related to owner gender-dog sex combination. F/f = female owner with female dog, F/m = female owner with male dog, M/m = male owner with male dog, M/f = male owner with female dog. Median, interquartile range, upper and lower whiskers and outliers are given.

The owner’s individual coefficient of variation of cortisol (iCV) did not correlate with the dog’s iCV (Spearman’s: *n* = 105, *r*_*s*_ = 0.043, *p* = 0.663). Female owners with male dogs had the lowest iCV of all other owner gender-dog sex combinations (*p<*0.001; Table 3, Fig 2). The higher the owner scored on the NEO-FFI dimension “Agreeableness” the higher the owner’s iCV (*p* = 0.028; Table 3). Also, owners high on the PCA-axis “aversive interaction style” had high iCV values (*p* = 0.043; Table 3). As a trend, owners with a cool and friendly dog (dog personality PCA-axis) had high iCV values (*p* = 0.083; Table 3).

**Table 3:** Effects of owner gender-dog sex combination, owner and dog personality, as well as interaction style on owner cortisol coefficient of variation (iCV) over the first and second session.

Explanatory Variable	*Df*	*F*	*p*
Owner gender-dog sex combination	4	14.194	**<0.001**
Owner Agreeableness (NEO-FFI Dimension 4)	1	4.981	**0.028**
Aversive interaction style (Interaction PCA-axis 2)	1	4.187	**0.043**
Dog cool-friendly (MCPQ-R PCA-axis 5)	1	3.068	0.083

GLM with “owner iCV” as dependent variable, n = 109, adjusted R^2^ = 0.872. Significances p<0.05 are given in bold.

The higher the owners scored on the NEO-FFI dimension Neuroticism, the lower their dogs’ iCV (*p* = 0.041; Table 4). As a trend, the owner personality NEO-FFI dimension Openness was related to a high iCV in dogs (*p =* 0.069; Table 4). The more the owner was insecure-ambivalently attached towards the dog (FERT PCA-axis 4), the lower the dog’s iCV (*p* = 0.016; Table 4). Owner relationship towards other humans was related to the dog’s iCV insofar as owners with separation anxiety towards other humans and who wished to be independent from other humans (RSQ axis 1 and 4, overall indications for an insecure attachment style with humans) had dogs with a low iCV (*p* = 0.007 and *p* = 0.003; Table 4). As a trend, owners with a lack of trust towards other humans (RSQ axis 3) had dogs with low iCV values (*p* = 0.068; Table 4).

**Table 4:** Effects of owner personality, owner to dog attachment and owner relationship to other humans on dog individual cortisol coefficient of variation (iCV) over the first and second session.

Explanatory Variable	*Df*	*F*	*p*
Owner Neuroticism (NEO-FFI Dimension 1)	1	4.290	**0.041**
Owner Openness (NEO-FFI Dimension 3)	1	3.366	0.069
Owner insecure-ambivalent (FERT PCA-axis 4)	1	5.948	**0.016**
Owner separation anxiety (RSQ axis 1)	1	7.661	**0.007**
Lack of trust (RSQ axis 3)	1	3.399	0.068
Owner wish to be independent (RSQ axis 4)	1	9.192	**0.003**

GLM with “dog iCV” as dependent variable, n = 115, adjusted R^2^ = 0.648. Significances p<0.05 are given in bold.

## Discussion

We investigated cortisol variability in human-dog dyads and hypothesized that high cortisol variability may be an indicator of efficient and adequate coping strategies. As expected, high cortisol variability was related to low absolute cortisol concentration values in owners and partly also in dogs. Furthermore, high cortisol variability was related to cortisol decrease in owners during the PVT and in dogs during the second threat, which supports our hypothesis. Cortisol variability (iCV) seems to follow the same principle as heart rate variability (HRV); heart rate (HR) also correlates negatively with heart rate variability. High HR indicates arousal, whereas high HRV indicates relaxation, good well-being and health status. HRV reflects the ability of the heart to adapt to changing circumstances [38]. Decreased cortisol variability is, similar to low HRV, related to bad health status and well-being [39–41]. This is supported by the psychobiological factors affecting dyadic HPA modulation, notably dyadic sex/gender combination, personality and attachment style.

Owners scoring high in Neuroticism had dogs with low cortisol variability. Neuroticism is linked to low expectations that social support will be forthcoming when needed [55], to major depression [85–87] and to anxiety [55,56]. Dogs are sensitive to their owners´ emotional states [25–29] and emotional contagion between owners and dogs is possible [32,33]. Thus, dogs may mirror the anxiety and negative expectations of neuroticistic owners in their cortisol variability. Interestingly, cortisol increase after stressful stimuli and also morning cortisol concentration are low in dogs from owners scaling high in Neuroticism [47,57]; but the opposite effect was found for cortisol variability in dogs. Cortisol reactivity may be more influenced by immediate interactions, e.g. dogs of neuroticistic owners approach them more often [62], which may dampen the immediate stress response. In contrast iCV, reflecting the regulatory capacity of the HPA axis, may be more integrative over time and contexts.

Highly agreeable people are known to cope better with the challenges of daily life [55] than less agreeable ones. Agreeable owners are probably good at coping with social situations and hence at controlling social stress. This personality dimension is described as being cooperative, sympathetic and considerate. Thus, agreeable owners had higher cortisol variability then less agreeable ones, indicating efficient stress coping.

Dog personality, as rated by owners, did not significantly explain cortisol variability in dogs and owners. In fact, owner characteristics seem to be more relevant in this respect [47,57,88] than just the characteristics of the dog itself. Still, perceiving one´s own dog as cool and friendly may feed back to the psychophysiological variability in owners, as it is probably easier to handle daily life with a relaxed dog, which in turn depends mainly on the performance of the human partner. In general, relaxed owners are likely to have relaxed and friendly dogs.

As predicted, owners’ insecure-ambivalent attachment towards their dog was related to low cortisol variability in dogs. Attachment classification is known to predict sensitivity and reliability as a caregiver [89,90]. Sensitive caregivers interact thoughtfully with the child and are aware of the child’s needs [89,90]. Thus, owners low in sensitivity may be more likely to have dogs with an insecure attachment style than owners with a sensitive caregiving style [91]. Dogs of the former are more stressed during an attachment assessment than securely attached dogs, and their owners are more likely to be insecurely attached towards them [47].

Our findings confirm previous results that the owner’s relationship towards other humans is reflected in the owner-dog relationship [92]. Attachment-related interaction styles, such as contact-seeking, are displayed in interactions with the dog in the same way as they are expected to be in interaction with humans [93]. Thus, the attachment representation with the primary human caregiver may also be transferred to the dog’s cortisol variability.

While gender is important, particularly the dyadic gender combination appears to influence social interactions in humans [48–51]. Men tend to show comparatively higher cortisol increases when their social prestige is challenged (e.g. by performing in front of an audience), whereas experiencing social rejection seems to be a more powerful trigger for HPA responses in women [94]. Furthermore, women report more fear and less happiness than men after a social stress test [95]. Men and women differ not just in their social emphases and coping strategies, but also in their interactions with, and attitudes towards, animals [44]. Women show stronger emotional relationships with companion animals than men do [46,96] and girls seek more contact with animals than boys [97]. During domestication, dogs became highly adapted to living with humans; therefore minor variations in the owners´ interaction styles may have distinct effects on the dogs´ physiological and behavioral responses (45). Dogs can probably discriminate human gender and may adapt their behavior according to the owner gender. For example, male dogs of female owners are less sociable and relaxed than male dogs of male owners [46]. This seems to be reflected in our result where female owners with male dogs were shown to have the lowest cortisol variability compared to all other dyadic gender combinations.

As expected, we found considerable individual cortisol variability for owners and dogs over the different experimental challenge situations. Interestingly, a decrease of cortisol over both sessions could be observed. This may reflect the recovery from travel and apprehension of a novel situation with subsequent relaxation after the initial phase; pretest baseline samplings are sensitive to the novelty of the setting and an adaptation to this setting may lead to lower samples afterwards [71]. Furthermore, we did not find a clear increase in cortisol in owners and in dogs during most of the tests, probably because they were predictable and controllable. The owners’ and dogs’ emotional support for each other could also be an explanation for the decrease of cortisol in some individuals during both sessions. For logistic reasons we were not able to control how dyads reached the lab and whether this may influence cortisol concentrations. But we figure that this was in alignment with the routines of each dyad and we do not consider the lack of this kind of control as crucial for interpreting the outcome of our study. In future studies a longer initial phase to adapt to the lab and a control group of dogs being regularly at the lab could prevent this limitation. We also acknowledge that a still higher sample size per group would increase statistical power and may lead to more distinct results on group level.

We conclude that individual cortisol variability over different challenging situations may be indicative of individual adaptation in humans as well as dogs. High intra-individual cortisol variability could be an indicator of efficient and adaptive coping, because a flexible HPA axis supports adequate responses to a variable social environment. We suggest that both owner and dog social characteristics influence dyadic cortisol variability, with the human partners being more influential than the dog. These findings underscore the importance of considering the human-dog dyad on a systemic level, i.e. including the social context in experimental science as well as in dog training and dog behavior therapy.

## Supporting Information

S1 Table Dataset. Data used for statistical analysis.
(XLSX)
